# An *in vitro* model system for testing chemical effects on microbiome-immune interactions – examples with BPX and PFAS mixtures

**DOI:** 10.3389/fimmu.2024.1298971

**Published:** 2024-06-17

**Authors:** Florence Fischer, Arkadiusz Pierzchalski, Sarah Riesbeck, Alix Sarah Aldehoff, Victor Alfonso Castaneda-Monsalve, Sven-Bastiaan Haange, Martin von Bergen, Ulrike Elisabeth Rolle-Kampczyk, Nico Jehmlich, Ana Claudia Zenclussen, Gunda Herberth

**Affiliations:** ^1^ Department of Environmental Immunology, Helmholtz Centre for Environmental Research – UFZ, Leipzig, Germany; ^2^ Department of Molecular Systems Biology, Helmholtz Centre for Environmental Research – UFZ, Leipzig, Germany; ^3^ Perinatal Immunology, Medical Faculty, Saxonian Incubator for Clinical Translation (SIKT), Medical Faculty, University of Leipzig, Leipzig, Germany

**Keywords:** microbiome-immune interaction, immune cell activation, T cells, bioreactor, microbial community, chemical mixtures, bisphenols, PFAS

## Abstract

**Introduction:**

More than 350,000 chemicals make up the chemical universe that surrounds us every day. The impact of this vast array of compounds on our health is still poorly understood. Manufacturers are required to carry out toxicological studies, for example on the reproductive or nervous systems, before putting a new substance on the market. However, toxicological safety does not exclude effects resulting from chronic exposure to low doses or effects on other potentially affected organ systems. This is the case for the microbiome-immune interaction, which is not yet included in any safety studies.

**Methods:**

A high-throughput in vitro model was used to elucidate the potential effects of environmental chemicals and chemical mixtures on microbiome-immune interactions. Therefore, a simplified human intestinal microbiota (SIHUMIx) consisting of eight bacterial species was cultured *in vitro* in a bioreactor that partially mimics intestinal conditions. The bacteria were continuously exposed to mixtures of representative and widely distributed environmental chemicals, i.e. bisphenols (BPX) and/or per- and polyfluoroalkyl substances (PFAS) at concentrations of 22 µM and 4 µM, respectively. Furthermore, changes in the immunostimulatory potential of exposed microbes were investigated using a co-culture system with human peripheral blood mononuclear cells (PBMCs).

**Results:**

The exposure to BPX, PFAS or their mixture did not influence the community structure and the riboflavin production of SIHUMIx *in vitro*. However, it altered the potential of the consortium to stimulate human immune cells: in particular, activation of CD8^+^ MAIT cells was affected by the exposure to BPX- and PFAS mixtures-treated bacteria.

**Discussion:**

The present study provides a model to investigate how environmental chemicals can indirectly affect immune cells via exposed microbes. It contributes to the much-needed knowledge on the effects of EDCs on an organ system that has been little explored in this context, especially from the perspective of cumulative exposure.

## Introduction

The high standard of living and comfort of everyday life in industrialized societies is based in part on the use of novel substances and chemicals. They help to increase crop yields, make food last longer, produce durable everyday items, make housework easier and turn personal hygiene into a wellness experience. However, in addition to these everyday benefits, it has been clear for some time that some of these chemicals can be potentially hazardous to health. Endocrine disrupting chemicals (EDCs), for examples, can interfere with hormone pathways and affect the human physiology.

Globally, the marketing of new chemicals is governed by regulations such as European Union’s (EU) REACH Regulation (Regulation (EC) No 1907/2006). The regulation requires manufacturers to carry out tests to assess the risk of a new substance to humans, animals and the environment. These tests aim to clarify the acute, potentially toxic potential of the substances but do not address the impact of chronic exposure to low doses of the chemicals. Tests are available for skin and eye irritation, genetic toxicology, acute toxicity, reproductive and developmental toxicity, carcinogenicity and endocrine disruption (Regulation (EC) No 1907/2006, Annexes VII-X). However, there are other biological targets that may be at risk from chemicals in our environment that are not yet included in the REACH regulation. For example, a rather new, but highly relevant perspective of the risk assessment is the evaluation of their effects on the gut microbiota (1, 2).

Gut microbes fulfill multiple physiological functions for their host, almost like an extra organ (3). For example, they provide colonization resistance to pathogens, ensure the energy supply to gut epithelial cells, communicate with the mucosal immune system and maintain gut homeostasis (4). Particularly in early life, the microbiota shapes the immune system and ‘teaches’ it to strike a balance between fighting potentially harmful pathogens and tolerating benign commensals and dietary antigens (5). On the other hand, like any other organ, the microbiota can become dysfunctional and cause adverse health effects in the host. So-called *dysbiosis* refers to any change in the microbiota compared to the microbiota of a healthy person, including shifts in community structure, function, or overall diversity (6). This condition has been implicated in the pathogenesis of several immune-related diseases, such as chronic inflammation, atopy and even cancer (7).

There is increasing evidence that certain environmental chemicals have adverse effects on the microbiota and thus indirectly on health (8). However, most of these studies have been conducted in mouse models. Therefore, extrapolation to humans is not always possible, particularly due to species differences (8). Another challenge in microbiota research is to establish causal links between dysbiosis and specific health outcomes. One possibility are germ-free or gnotobiotic animals, in which the microbiota can be specifically manipulated (9). These experiments are complex, time- and cost consuming and not feasible on a large scale. However, assessing the harmful potential of environmental chemicals on host-microbe interactions would require such assays, which would also need to be standardized, reproducible and feasible on a high-throughput scale. Here, we present a model that fulfills these criteria and characterizes the effects on the structure and functions of the human gut microbiota and resulting health outcomes. Two groups of EDCs, namely bisphenols (BPX) and per- and polyfluoroalkyl substances (PFAS), were selected as representative of widely used environmental compounds to determine the functionality and applicability of the model for chemical testing.

BPX are used in the manufacture of polycarbonate plastics and epoxy resins and can be are found in many consumer products including food packaging and storage boxes, food cans and plastic bottles. Bisphenol A (BPA; 2, 2-bis [4-hydroxyphenyl] propane) has been the most widely used BPX (10). However, due to its endocrine disrupting properties, BPA was classified as a substance of very high concern (SVHC) under the REACH Regulation (ED/01/2017) and its industrial use was more strictly regulated. In fact, BPA was already banned in 2011 in the European Union (EU; EU directive 2011/8/EU) for the manufacture of polycarbonate baby bottles. Thus, BPA is increasingly being replaced by analogoue chemicals such as bisphenol F (BPF, 4-[9-(4-hydroxyphenyl)-9H-fluoren-9-yl]phenol) and bisphenol S (BPS; 4,4’-sulfonyldiphenol) (11). Consequently, while serum levels of BPA have tended to decrease in recent years, BPF and BPS are more prevalent and detectable at higher concentrations, i.e. around 0.3 ng/mL in human serum (12).

PFAS are used in many industrial processes and consumer products due to their special technical properties including water and grease repellency, chemical and heat stability. They are classified as persistent organic pollutants (POPs) and have been found in soil and drinking water around the world. The more than 10,000 different substances that make up this family of chemicals can be distinguished by the length of their carbon chain: while perfluorohexane acid (PFHxA) and perfluorobutanoic acid (PFBA) are classified as short-chain PFAS, perfluorooctanoic acid (PFOA) has a long carbon chain. Chain length determines among other things, the biological effects of a given substance, including accumulation in body compartments and binding affinity to proteins (13–15). Concentrations of individual PFAS in human serum are mostly between 0.4 ng/ml and 10 ng/ml, with perfluorooctane sulfonic acid (PFOS) and PFOA being the most abundant (16–18).

Previously, we showed that exposure to environmentally relevant concentrations of individual BPXs affected the structure and function of a human gut microbial consortium (19, 20). We also found changes in their immunomodulatory properties, specifically their potential to modulate the activation of human mucosal-associated invariant T (MAIT) cells (20). This innate-like CD8^+^ lymphocyte population is critical for maintaining gut homeostasis, i.e. antibacterial host defense and tissue repair (21). Interestingly, unlike conventional T cells, the activation of MAIT cells is not restricted by MHC class 1 and 2. Instead, they are able to recognize microbial metabolites presented via MHC class 1-related protein 1 (MR1), with bacteria-derived riboflavin metabolites promoting and folate derivates inhibiting MAIT cell activation (22–24). Upon stimulation, they upregulate the expression of the activation marker CD69, the degranulation marker CD107a and the production of the pro-inflammatory cytokines TNF-α and IFN-γ.

While few studies have analyzed the effects of individual BPX and PFAS, the effects of chemical mixtures on microbiome-immune interactions have been less assessed, but are undoubtedly important for health risk assessment. The aim of the present study was to establish a model that reveals the mutual influence of different chemicals, i.e. whether they can enhance or attenuate each other’s biological effects on gut microbes and consequently alter their potential to stimulate human immune cells. Therefore, we cultured the extended simplified human intestinal microbiota [SIHUMIx, (25, 26)] *in vitro* by using a continuous flow cultivation system and treated the microbes with mixtures of BPX (BPF, BPS), PFAS (PFBA, PFHxA, PFOA) or the combination of both chemical classes. During the course of cultivation, microbial samples were taken to 1) to analyze the effects of the chemicals on the microbiota structure and riboflavin metabolism and 2) to investigate alterations of the immune activating potential of the bacteria, particularly at the level of T cell subpopulations (e.g. MAIT cells).

## Methods

### Simplified human intestinal microbiota – SIHUMIx

The extended simplified human gut microbiota (SIHUMIx) comprises of eight species including *Anaerostipes caccae* (DSMZ 14662), *Bacteroides thetaiotaomicron* (DSMZ 2079), *Bifidobacterium longum* (NCC 2705), *Blautia producta* (DSMZ 2950), *Clostridium butyricum* (DSMZ 10702), *Clostridium ramosum* (DSMZ 1402), *Escherichia coli K-12* (MG1655) and *Lactobacillus plantarum* (DSMZ 20174) (25). These bacterial strains were chosen based on their abundance in the human intestine, the spectrum of fermentation products and community stability in *in vitro* culture (26). The cultivation protocol, the growth conditions and media ingredients were used as described earlier (26).

### Experimental set-up

**Figure 1:**
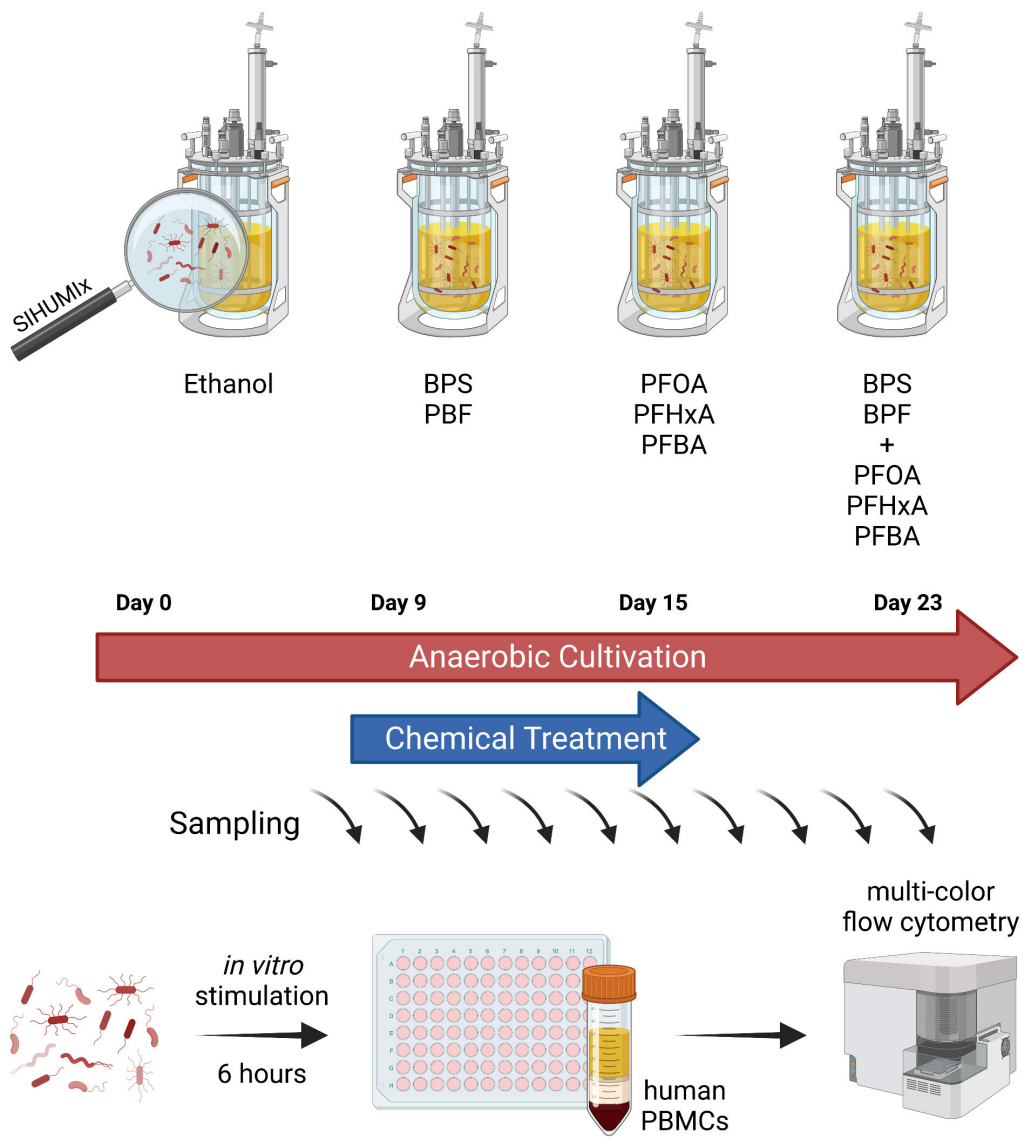
Experimental model to investigate effects of EDCs on host-microbe-interactions. SIHUMIx were cultured in a continuous flow cultivation system. From day 9 to 15 of cultivation, mixtures of BPX (BPF, BPS; each 11 µM), PFAS (PFBA, PFHxA, PFOA; each 1.33 µM) or the combination of both chemical classes were added to the bacterial community, ethanol was used as solvent control. Samples were taken at several time points for metaproteome analysis, riboflavin measurement and immune stimulation. For the latter, bacterial cells were washed and fixed prior to addition to human PBMCs for 6 hours. The effects of EDC-treated SIHUMIx on immune cell activation was analyzed by multi-color flow cytometry. SIHUMIx, extended simplified human intestinal microbiota; BPX, bisphenols; PFAS, perfluoroalkyl and polyfluoroalkyl substances; EDC, endocrine disrupting chemicals. The figure was created with Biorender.

Continuous flow cultivation of SIHUMIx was carried out in an *in vitro* bioreactor system (Multifors 2 chemostat, Infors, Switzerland). The reactors were filled with a human complex intestinal medium (CIM) as described by McDonald et al. (27) and modified as reported in (19) at day -1. The medium turnover was set to 250 ml per 24 h, pH was maintained at 6.5 and redox potential was monitored throughout the experiment to ensure anaerobic conditions. Two weeks before the start of the bioreactor experiment the SIHUMIx strains were freshly thawed from glycerol stocks individually in Brain-Heart-Infusion (BHI) as reported in (19). On the day of inoculation (day 0), the cell number from over-night cultures of each strain was calculated based on OD600 (cell number = OD600 × 8*10^8^). Each bioreactor (250 ml) was inoculated with a total of 8*10^9^ cells (1*10^9^ of each strain). The continuous flow was started after 24 h of inoculation. The bioreactor experiment was composed of four phases: (1) a sterile run with CIM medium only (d -1 to d0), (2) a community stabilization and adaptation phase (day 0 to day 8), (3) a chemical exposure phase (d9 to d16) and (4) a recovery phase (d17 to d23) (**Figure 1**).

### Chemical treatment

All chemical stocks were prepared in ethanol. We tested three chemical conditions: (1) PFOA, PFHxA and PFBA (Sigma) (1.33 µM each) were mixed prior to treatment to achieve a 4 µM PFAS stock solution, a concentration within a range which has been observed to be ingested by occupationally exposed adults (28, 29). (2) BPF and BPS (Sigma Aldrich, St. Louis, USA) in a concentration of 11 µM each were mixed prior to treatment and hereafter termed as BPX. This concentration refers to an environmental realistic uptake calculated based on urine concentrations and the tolerable daily intake (TDI) of BPA from 2015, ranging between 4 to 5 µg/kg bw per day (10, 30). Calculations are provided in **Supplementary Material**, **Supplementary Table 2**. (3) A mix of our PFAS and BPX stocks. Control reactors were treated with ethanol alone. The final ethanol concentration in all reactors was 0.2%. Exposure started from day 9 and the initial dose of chemicals contained either 4 µM PFAS, 22 µM BPX or 4 µM PFAS + 22 µM BPX. Subsequent treatments contained half the initial dose of each chemical and were added every 12 h. All treatments were directly added into the bioreactor vessel via an inoculation port.

### Sampling

Bioreactors were sampled for metaproteome and riboflavin measurements at day 1, 8, 9, 16, 18 and 23. Bacterial cultures (2 ml) per reactor were centrifuged at 5000 × g at 4°C for 10 min. The supernatant was transferred to a separate tube for targeted riboflavin measurements, while the bacterial cell pellet was stored at -80°C for subsequent metaproteomic sample preparation.

Bacterial cultures (2 ml) for immune cell stimulation were harvested at day 7, 10, 15 and 23 by centrifugation (3.200 g, 5 min, 4°C) and fixed by resuspension in 1% of formaldehyde for 1 min. Bacterial cells were washed three times with phosphate buffered saline (PBS, 140 mM NaCl, 10 mM Na2HPO4, 7 mM KH2PO4). The cell number was determined using a Beckman Coulter Multisizer 3 cell counting system (Beckman Coulter, Brea, USA) and adjusted to 3 x 10^9^ cells/mL in IMDM medium (IMDM supplemented with 10% fetal calf serum, 25 mM HEPES, 50 µM β-Mercaptoethanol and 100 U/mL Penicillin/Streptomycin). Bacterial pellets were stored with supernatant at -80°C.

### Riboflavin analysis

#### Sample preparation

Riboflavin was extracted with 5 volumes of a mixture of methanol/acetonitrile/water (2/3/1) and 10 µL internal standard added. After addition of 5 volumes of extraction solvent, samples were vortexed for 5 min and sonicated in an ultrasound bath for additional 5 min. After centrifugation at 14000 rpm for 5 min the supernatant was transferred to a fresh tube and dried in a SpeedVac™ vacuum concentrator (Eppendorf, Hamburg, Germany). The dried extract was resuspended in 100 µL of a mix of running solvent A and running solvent B (1:1).

### Measurement and data analysis

For LC-MS/MS measurement 10 µL of the resuspended extract were injected into a HPLC-MS-System (RSLC Ultimate 3000 Thermo Fisher coupled with Q-Trap 5500 AB Sciex). Metabolites were separated on ACQUITY UPLC BEH 300 C18 (1,7 µm, Waters) with a flow rate of 0.3 mL/min with gradient of running solvent A (0.1% formic acid in water) and running solvent B (0.1% formic acid in methanol): start with 100% B and holding over 5 minutes, minutes 5 to seven 99% B and ending from 7 to 10 minutes with 1% B. The Q-Trap was set up to positive MRM mode Riboflavin MRM: parent ion: 377product ions: 243, 198, and 172, Internal Standard MRM: parent ion: 383 product ions: 249, 202, and 175. Quantification was performed with Analyst Software (1.7.1).

### Proteome analysis

For protein analysis, samples were dissolved in lysis buffer (8 M Urea, 2 M Thiourea, 1 mM PMSF). Protein extraction was done by incubation at 95°C, 1,400 rpm and 5 min, afterwards by 3 min ultrasonication water bath. To the pellets, 6.75 µL 2,5 mM 1,4 dithiothreitol (in 20 mM ammonium bicarbonate) were added and incubated for 1 h at 60°C and 1,400 rpm shaking. Afterwards, 150 µL 10 mM iodoacetamide (in 20 mM ammonium bicarbonate) were added and incubated for 30 min at 37°C and 1,400 rpm shaking in the dark. Finally, 200 µL of 20 mM ammonium bicarbonate were added and the protein lysates were proteolytically cleaved overnight at 37°C with trypsin (2.5 µL of 0.1 µg/µl trypsin, Promega). The cleavage was stopped by adding 50 µL 10% formic acid. The peptide lysates were desalted using ZipTip μC18 tips (Merck Millipore, Darmstadt, Germany). The peptide lysates were re-suspended in 15 µL 0.1% formic acid and analyzed by nanoliquid chromatography mass spectrometry (UltiMate 3000 RSLCnano, Dionex, Thermo Fisher Scientific). Mass spectrometric analyses of eluted peptide lysates were performed on a Q Exactive HF mass spectrometer (Thermo Fisher Scientific) coupled with a TriVersa NanoMate (Advion, Ltd., Harlow, UK). Peptide lysates were injected on a trapping column (Acclaim PepMap 100 C18, 3 μm, nanoViper, 75 μm x 2 cm, Thermo Fisher Scientific) with 5 μL/min by using 98% water/2% ACN 0.5% trifluoroacetic acid, and separated on an analytical column (Acclaim PepMap 100 C18, 3 μm, nanoViper, 75 μm x 25 cm, Thermo Fisher Scientific) with a flow rate of 300 nL/min. Mobile phase was 0.1% formic acid in water (A) and 80% ACN/0.08% formic acid in water (B). Full MS spectra (350–1,550 m/z) were acquired in the Orbitrap at a resolution of 120,000 with automatic gain control (AGC) target value of 3×106 ions.

Acquired LC-MS data were analyzed with the Proteome Discoverer (v.2.5, Thermo Fischer Scientific) using SEQUEST HT and INFERYS Rescoring. Protein identification was performed using a database (SIHUMIx.fasta and common contaminations). Searches were conducted with the following parameters: Trypsin as enzyme specificity and two missed cleavages allowed. A peptide ion tolerance of 10 ppm and an MS/MS tolerance of 0.02 Da were used. As modifications, oxidation (methionine) and carbamidomethylation (cysteine) were selected. Peptides that scored a q-value >1% based on a decoy database and with a peptide rank of 1, were considered identified.

### Isolation of human PBMCs

Buffy coat samples from six healthy, male volunteers were obtained from the blood donation service at the University Hospital of Leipzig, Germany. The study was approved by the Ethics Committee of the University of Leipzig (Ref. 079–15-09032015). All participants had given written informed consent. Peripheral blood mononuclear cells (PBMC) were isolated from the buffy coats by density-gradient centrifugation using Ficoll-Paque Plus (GE Healthcare, Little Chalfont, UK). Cells were gradually frozen in FCS containing10% DMSO at -80°C and stored at -150°C until they were used for *in vitro* stimulation assays.

### *In vitro* stimulation of human PBMCs with chemical-treated bacteria

Human PBMCs were stimulated with bacteria *in vitro* as described before (26, 31). Briefly, the PBMCs were cultured for 24 hours at 10^6^ cells/well (respectively 500,000 cells/well for positive and negative control) in IMDM (GlutaMax supplement, Fisher Scientific, Schwerte, Germany) supplemented with 10% fetal bovine serum (FBS, Biochrom, Berlin, Germany), 1 X Pen-Strep Solution (Biowest, Nuaillé, France), and 50 µM β-mercaptoethanol (AppliChem, Darmstadt, Germany) in a 96-well U-bottom microplate (Greiner Bio-One, Frickenhausen, Germany) at 37°C in a 5% CO_2_ incubator. Thereafter, PBMCs were stimulated with SIHUMIx bacterial consortium at 25 bacteria per cell (bpc). *E. coli* (20 bpc) was used as a positive control. Supplemented IMDM was used as negative/unstimulated control. After 2 hours of incubation at 37°C in a 5% CO_2_ incubator, 1 µM monensin and 10 µg/ml brefeldin A were added to the culture and the cells were incubated for another 4 hours. Samples were run in triplicates.

### Flow cytometry

To exclude dead cells, Zombi NIR™ viability dye (BioLegend, 1:3,000) was used prior incubation with antibodies for cell surface staining (**Supplementary Table 1**) for 30 min at room temperature (RT). As it is known that α-CD107a (1:1,800) may be internalized during *in vitro* stimulation, the antibody was already added to the cells after 2 hours of *in vitro* stimulation with bacteria. Afterwards, the cells were fixed in FACS™ lysing Solution (BD) and permeabilized using FACS™ Permeabilizing Solution 2 (BD Biosciences, San Jose, CA) according to manufacturer’s instruction. For intracellular staining, cells were incubated with antibodies against cellular activation markers for 20 min at RT. Flow cytometric analysis was performed by using 3L Cytek^®^ Aurora full spectrum flow cytometer (Cytek Biosciences, California, US). A minimum of 150,000 viable cells per sample have been acquired.

Flow cytometry data were analyzed with FCS Express 7 (*De Novo* Software). Flow cytometry data were pre-processed as follows: fluorescence data were scaled and checked for uniform positive and negative events expression. Then by using FlowCut, a New Low Time Density Downsampling was set as 0.1. In New Magic Number Downsampling the outliers definition segment size was set to 500 and max % of events that may be marked as outliers was set to 30. An in-depth analysis for high dimensional data was performed via t-stochastic neighbor embedding (t-SNE) analysis for the expression of cell surface markers. Gate downsampling was set to alive cells, sample size to 5000 events (all together 120000 events from 24 files being merged for t-SNE). The t-SNE method used was Barnes-Hut approximation with an amount of approximation of 0.50, perplexity of 30 and 500 iterations and Opt-SNE was used. Distinct populations were identified via manual gating supported by the expression of cell surface markers. A manual gating strategy was applied to emphasize specific immune cell populations including B cell, CD4 T cells (Th2 and Th17 cells), CD8 T cells, Tfh cells, NK cells, iNKT cells, MAIT cells, γδ T cells and NKT cells, on the plot. Due to their reactivity to microbiota, particularly MAIT cells were on our focus and identified via manual gating as CD8^+^ CD4^-^ or CD8^-^ CD4^-^ CD3^+^, CD161^+^ and TCRVα7.2^+^ cells as shown in **Supplementary Figure 1**. A fluorescens minus five control (FM5) for all activation markers was used to identify positive cell populations.

### Statistical analysis

The data were analyzed with GraphPad Prism 9. Normal distribution was tested by Kolmogorov-Smirnov test. Significance was calculated by using the tests indicated in figure legends including PERMANOVA, Friedman test followed by Dunn’s multiple comparison test and RM-One-Way ANOVA with Geisser-Greenhouse correction followed by Dunnett’s multiple comparison test. Values less than 0.05 were considered statistically significant.

## Results

### Effect of chemical treatment on SIHUMIx community structure and riboflavin production

Bioreactors were inoculated with pre-cultured bacteria. After a stabilization phase of eight days, BPX (each 11 µM), PFAS (each 1.33 µM), the mixture of both or the vehicle substance (ethanol) were added to the culture at run day nine. Half the initial dose of each substance was then added every 12 hours from day nine to day 15. Samples were taken at different time points to investigate the community structure by metaproteome analysis.

**Figure 2:**
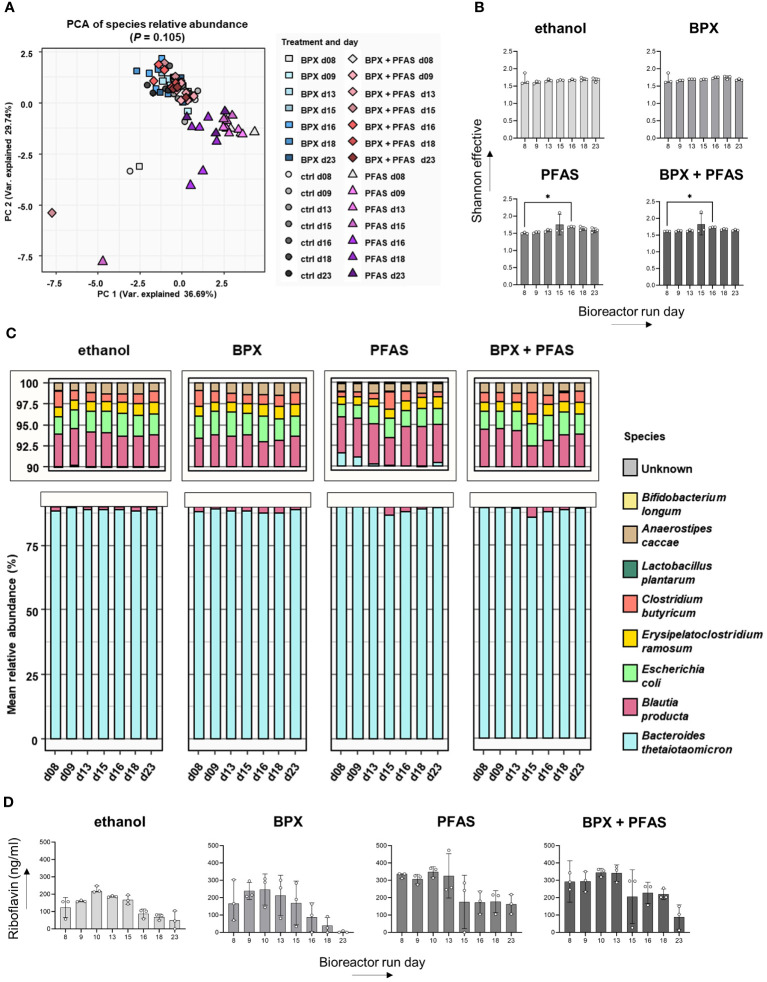
Effect of EDCs on SIHUMIx community composition and metabolism. SIHUMIx were cultured in bioreactors and exposed to BPX (22 µM) and/or PFAS (4 µM) or ethanol as solvent control from day 9 to day 15. Samples were taken at indicated time points for metaproteome analysis and riboflavin measurement. **(A)** Principal component analysis based on relative protein abundances shows abundance on several days and different chemical treatments. PERMANOVA p-value days; n = 3. **(B)** Alpha diversity shown as Shannon effective; n = 3; statistical test: Friedman test followed by Dunn’s multiple comparison test (in comparison to day 8). * P < 0.05. **(C)** Mean relative abundance of individual species in the course of days and according to chemical treatments; n = 3. **(D)** Riboflavin concentration in the culture supernatant measured by LC-MS/MS; n = 3; statistical test: Friedman test followed by Dunn’s multiple comparison test (in comparison to day 8).

Beta diversity analysis using PCA revealed that cultivation time, but not chemical treatment, significantly altered the community structure (**Figure 2A**). Furthermore, we found only minor effects of EDCs on alpha diversity, i.e. PFAS and BPX+PFAS resulted in increased Shannon diversity at bioreactor run day 16, 24 hours after the end of chemical treatment (**Figure 2B**). This is also reflected in **Figure 2C**, which shows the relative abundance of individual microbes of SIHUMIx over cultivation time as measured by metaproteome analysis (**Figure 2C**). The composition of the microbiota in the different bioreactors was very similar, although not completely identical. The analysis also confirmed the presence of the entire SIHUMIx consortium over the course of cultivation.

As the bacterial metabolite riboflavin specifically activates immune cells, we measured riboflavin in the SIHUMIx culture supernatant by LC-MS/MS. Despite the differences in absolute riboflavin concentration, the course of riboflavin concentration for each group showed an increase until day ten, followed by a decrease until the end of the bioreactor run (**Figure 2D**). We could not detect any significant change in riboflavin concentration as a result of the chemical treatment.

### Chemical treatment of SIHUMIx altered their immune cell activating potential

The gut microbiota exerts immunomodulatory effects either via direct bacterial-cell contact or via metabolites. It stands to reason that changes in the microbiota would be reflected in changes in the activity status of immune cells. Although we did not observe changes in microbial community structure after exposure to environmental chemicals, we wondered whether mixtures of BPX, PFAS or the combination of both would affect the immunostimulatory potential of SIHUMIx. We decided to focus initially on bioreactor run day ten, as this time point reflects 24 hours of chemical exposure when community adaptations may have occurred and riboflavin production was highest.

**Figure 3:**
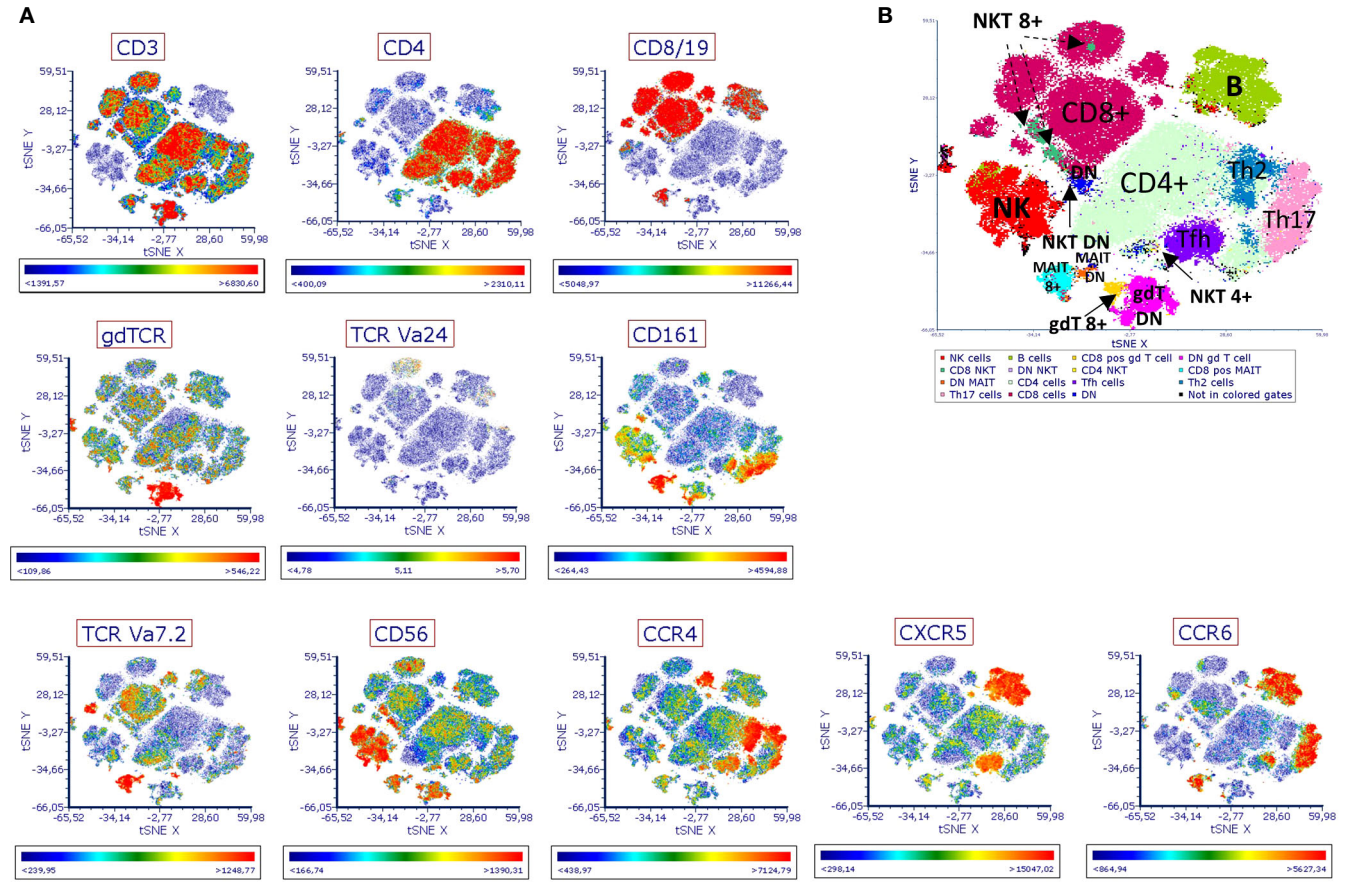
Immune landscape of human PBMCs stimulated with EDC-treated SIHUMIx. Human PBMCs were stimulated for 6 hours *in vitro* with fixed SIHUMIx pre-exposed to BPX (22 µM) and/or PFAS (4 µM) or ethanol as solvent control for 24 hours (bioreactor run day 10). The presence of indicated cell surface markers was measured by flow cytometry and analyzed via t-SNE. **(A)** The distribution of indicated cell surface markers. Scale bars of each t-SNE plot show color coding of fluorescence intensity. **(B)** The identification of distinct population was guided by the expression of cell surface markers. DN, double negative for CD4/CD8 marker; MAIT 8+, for CD8 positive MAIT cells; NKT 8+ for CD8 positive NKT cells; gdt 8+ for CD8 positive gd T cells.

Bacterial cells collected from each bioreactor on day ten of the run were used to stimulate human PBMCs *in vitro.* The phenotype and activation status of different immune cell populations were then measured by flow cytometry. The antibody panel used in this study included twelve cell markers and five markers of immune activation (**Supplementary Table 1**). After manual exclusion of doublets and dead cells, t-stochastic neighbor embedding (t-SNE) analysis was applied to reduce dimensionality. A manual gating strategy was applied to highlight specific immune cell populations on the plot (**Figure 3**, **Supplementary Figure 1**).

**Figure 4:**
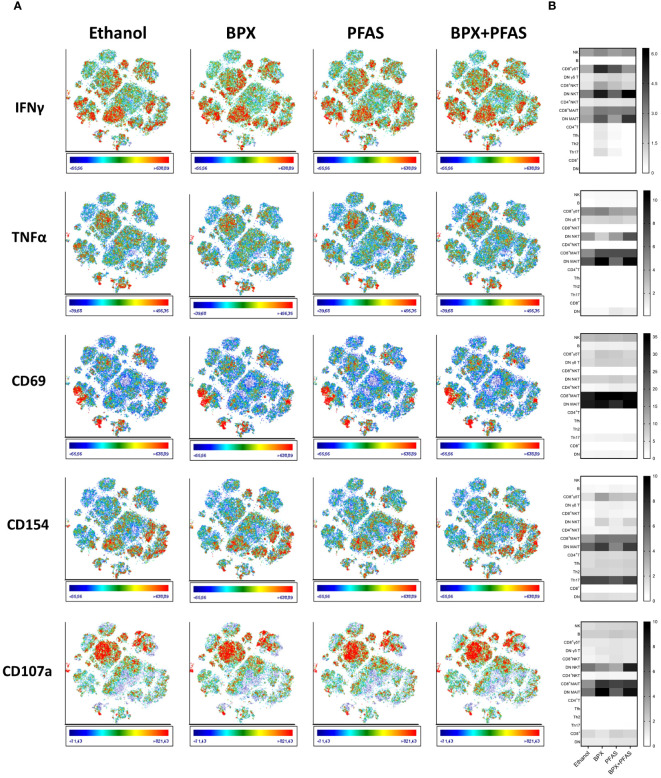
Activation of human immune cells stimulated with EDC-treated SIHUMIx. Human PBMC were stimulated for 6 hours *in vitro* with fixed SIHUMIx pre-exposed to BPX (22 µM) and/or PFAS (4 µM) or ethanol as solvent control for 24 hours (bioreactor run day 10). **(A)** The expression of indicated cell activation markers was measured by flow cytometry and is shown on t-SNE plots. Scale bars of each t-SNE plot show color coding of fluorescence intensity. **(B)** The relative expression of indicated cell activation markers (frequency of cells expressing the respective marker in %) was quantified for individual populations identified via t-SNE and is shown as heatmap.

Based on population density and the expression of immune cell markers, we identified 15 different populations on the t-SNE plot (**Figure 3B**, **Supplementary Figure 2**). Expression analysis of activation markers revealed group differences between several of the identified populations. SIHUMIx exposed to chemicals seemed to have an increased potential to activate immune cells of different population including CD8^+^ MAIT cells, DN MAIT cells (double negative, CD8^-^ CD4^-^ MAIT cells), NKT cells, CD8^+^ γδ T cells (**Figure 4**). Interestingly, this effect was more pronounced for some populations and markers (e.g. DN MAIT cells and DN NKT cells with IFN-γ, TNF-α and CD107a) when the bacteria were exposed to the mixture of chemical classes compared to SIHUMIx treated with a single class of chemicals, indicating that both chemical classes potentiate each other’s effect. On the other hand, the opposite was observed for other populations. The expression of most of the activation markers measured in CD8^+^ γδ T cells was mainly induced by stimulation with SIHUMIx exposed to either BPX or PFAS, but not to both classes of chemicals. Finally, there was some evidence of immune cell inhibition by PFAS-exposed microbes, as indicated by reduced expression of IFN-γ, TNF-α, CD154 and CD1078a in DN MAIT cells.

Thus, exposure of SIHUMIx to BPX or PFAS appears to alter the immune-activating potential of the bacteria. However, both the strength and direction of the effect, appear to depend on the particular cell population. Similarly, whether the combination of BPX and PFAS enhances or attenuates the immunostimulatory properties of the bacteria needs to be investigated in the context of the particular immune cell subtype.

### Chemical treatment of SIHUMIx modulated their MAIT cell activating potential

As observed in the t-SNE analysis, the two populations apparently activated by chemically treated SIHUMIx were the CD8^+^ MAIT and the DN MAIT cells. While both populations expressed similar levels of CD3^+^, CD161^+^ and TCRVα7.2^+^, they differed in their expression of CD4 and CD8 (**Supplementary Figure 2**).

To test the statistical significance of the findings from the t-SNE analysis, the expression of activation markers following *in vitro* stimulation of PBMCs with treated SIHUMIx, collected at run days 7, 10, 15 and 23, was quantified by flow cytometry for all individual blood donors and analyzed using a conventional, manual gating strategy (**Supplementary Figure 2**). Unfortunately, the DN MAIT cells are a very rare cell population within the PBMCs, so it was not possible to follow this particular population by conventional gating. Therefore, the following analysis focuses on CD8^+^ MAIT cells only.

As mentioned above, SIHUMIx established a stable consortium during the first six to eight days of *in vitro* cultivation in the bioreactor system. Nevertheless, differences between the four bioreactor groups (ethanol, BPX, PFAS, and BPX+PFAS) were still measurable after eight days of cultivation, especially for the absolute riboflavin concentrations in the culture supernatant. Therefore, we decided not to compare the four groups of bioreactors directly, but to analyze the groups of bioreactors individually over the course of cultivation. We used day 7 as starting point and tested whether the following chemical treatment affected the immune cell stimulating potential of the consortium compared to this reference time point.

**Figure 5:**
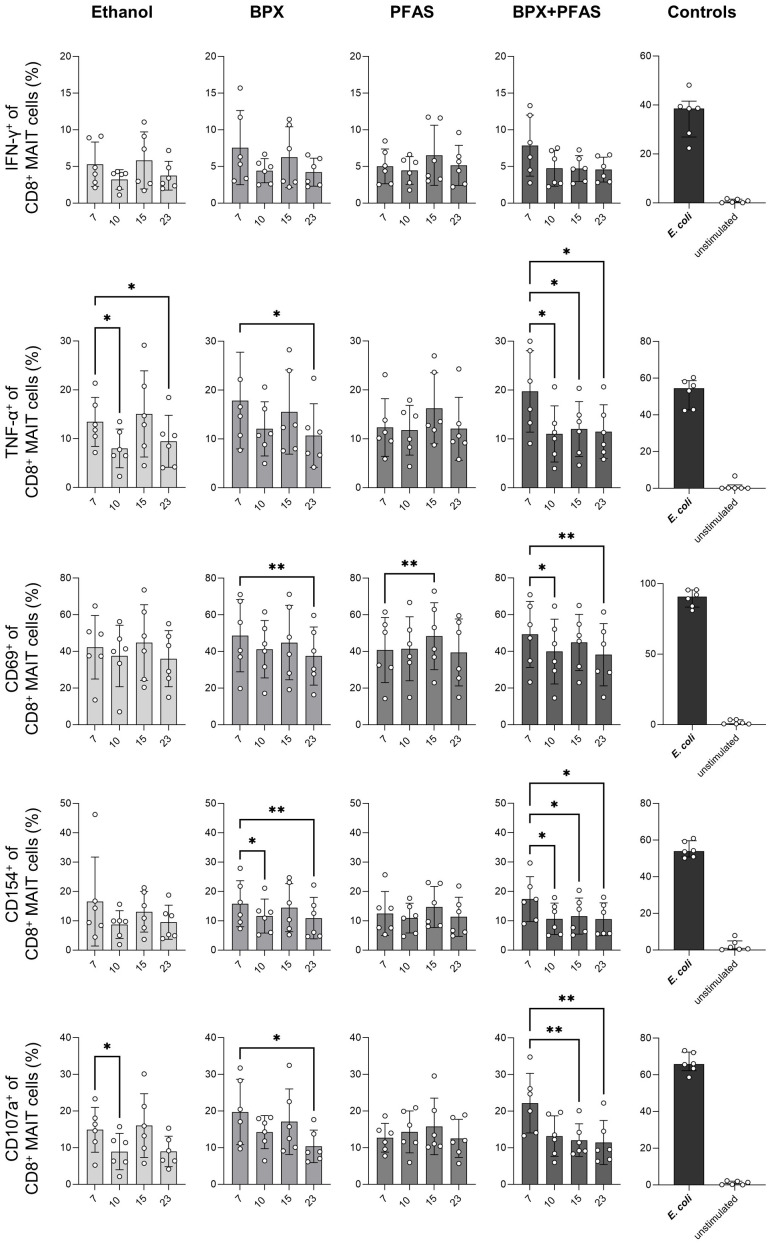
Activation of human CD8^+^ MAIT cells stimulated with EDC-treated SIHUMIx. The expression of indicated activation markers was measured by flow cytometry in human CD8^+^ MAIT cells after 6 hours *in vitro* stimulation of PBMC with ethanol- or chemical-treated SIHUMIx, all sampled at bioreactor run day 7, 10, 15 and 23. To test cell activation, untreated *E. coli* was used as a positive control and medium only was used as negative/unstimulated control. Shown are individual values representing different blood donors, means and SD. Statistical analysis: RM-One-Way ANOVA with Geisser-Greenhouse correction followed by Dunnett’s multiple comparison test (in comparison to run day 7); n = 6. * P < 0.05, ** P < 0.01.

Exposure of SIHUMIx to ethanol decreased the microbial potential to induce the expression of TNF-α (day 10 and 23) and CD107a (day 10) of CD8^+^ MAIT cells (**Figure 5**), indicating that the solvent already induced changes of the gut microbes. PBMCs stimulated with SIHUMIx, pre-exposed to BPX, showed similar activation characteristics, i.e. a decreased expression of TNF-α and CD107a (day 23). In addition, the ability of SIHUMIx to induce CD69 (day 10) and CD154 (day 10 and 23) expression by CD8+ MAIT cells was reduced by exposure to BPX (**Figure 5**). As changes in CD154 expression were only observed in the BPX-treated and not in the ethanol-treated group, this effect could indeed be induced by BPX. With regard to PFAS, the only significant effect observed was on CD69 expression after simulation with PFAS-treated SIHUMIx sampled on run day 15 (**Figure 5**). Interestingly, the mixture of BPX and PFAS induced most of the significant changes. BPX- and PFAS-treated SIHUMIx resulted in decreased expression of TNF-α, CD69, CD154, and CD107a from CD8^+^ MAIT cells compared to untreated SIHUMIx at most time points after chemical treatment (**Figure 5**, **Supplementary Figure 3**).

## Discussion

Interactions between commensal microbes and immune cells are critical for the development and function of the human immune system. Changes at the microbiota level, including shifts in community structure, function, or overall diversity can disrupt immune homeostasis and lead to immune-mediated diseases (32). Few studies have examined the influence of environmental chemicals on microbiome-immune interactions. However, most of these are *in vivo* studies, mainly performed with single chemicals (8), which do not necessarily reflect the situation in humans or the occurrence of chemical mixtures. Therefore, appropriate models are urgently needed for adequate risk assessment of environmental effects on microbiome-immune interactions. The aim of the present study was to establish a test system suitable for determining the relationship between environmental exposure, gut microbiota, and host immune effects in an easily standardized, high-throughput manner. To this end, we used a human microbiota model in a gut-like bioreactor system to track the effects of BPX, PFAS and their mixture on the microbial consortium. We then co-cultured the chemical-exposed gut bacteria with human immune cells in order to uncover a link to potential immunomodulatory effects.

Only in recent years, it has become evident that EDCs are among the most critical factors that can lead to microbiome disturbance, e.g. dysbiosis (8). A substantial number of studies focused particularly on BPA. They confirmed loss of diversity, changes in the community structure and alterations of the bacterial metabolome after exposure to this compound in mice and zebrafish (33–35). In addition, there are currently a few studies investigating the effects of other bisphenols on the gut microbiota. All of these studies suggest that in addition to BPA, its substitutes also have microbiota-disrupting effects (36–38). Notably, Wang and colleagues focused not only on individual substances, but also on the effects of a BPF/BPS mixture. They found that the individual compounds and the mixture resulted in different taxonomic changes, with BPS appearing to have the strongest effect on the microbiota (37). Taken together, these studies show firstly that it is questionable whether BPA substitutes are really less harmful alternatives and secondly that it is worthwhile including a mixture of chemicals in the experimental design as it is more likely to approximate actual exposure in the environment. In addition to BPX, other environmental chemicals have been the focus of microbiome studies, including PFAS. Evidence for their potential to disrupt the microbiota is already available from epidemiological studies (39). Furthermore, exposure to PFOA significantly altered the gut microbiota of mice, with an increase in Bacteroidetes and a decrease in fecal short-chain fatty acid (SCFA) concentrations, suggesting consequences of PFAS exposure at both the taxonomic and functional levels (40, 41).

Overall, both BPX and PFAS are potentially harmful to the gut microbiota. However, in the present study, however, neither mixtures of BPX or PFAS nor the mixture of both affected the community structure of SIHUMIx during the course of cultivation. This finding contrasts with the results of the *in vivo* studies mentioned above, which may due to several reasons. Firstly, the concentrations used in the present study reflect the human intake of BPX and PFAS, calculated on the basis of urinary and blood serum concentrations (10). It is not yet known how much of the ingested chemicals actually reach the colon, i.e. the gut microbiota. It is also unclear how long chemicals remain in the gut. In rats, orally administered BPA has been shown to persist in the gut after elimination from other tissues and plasma (42). Therefore, some EDCs may be expected to accumulate in the gut *in vivo*, exposing the microbiota to higher concentrations and/or for longer periods of time. The exposure time in the present study was only seven days, which may be a short time for the consortium to adapt. Another aspect that could not be reflected by the bioreactor model is the biotransformation of the chemicals by host’s xenobiotic enzymes of the host, which generate different metabolites. also It should be noted that microbes are also capable of several biotransformation processes (8). For both BPX and PFAS, several metabolites have been described that exhibit different biological activities than the original compound (43, 44). The effects of BPX and PFAS metabolites on gut microbes are not yet understood and require further investigation. Finally, SIHUMIx consists of eight representatives of the human gut microbiota (26). In a previous study, single bacterial strains *B. thetaiotaomicron* and *E. coli* and human fecal microbiota were exposed to BPX in a batch culture system. We found that sensitivity to EDCs differed between single bacteria and a complex consortium, as evidenced by differences in growth viability and microbial metabolism (20). A simplified microbial consortium cannot fully mimic a complex microbiota, which may explain the conflicting results between *in vitro* and *in vivo* studies.

Of note, there are two main ways in which environmental chemicals can affect the microbiota: (a) by altering the microbial taxonomy and diversity, and (b) by altering the functional phenotype of the microbiota (8, 45). Although no effects of EDCs on microbial community structure and riboflavin concentration were detected in the present study, we cannot conclude that BPX and PFAS do not cause other changes, i.e. functional alterations of the microbial metabolism, such as SCFA. Therefore, a global analysis of metabolites produced by SIHUMIx in the presence of environmental chemicals would improve our understanding and should be considered in future studies.

Despite the differences between *in vivo* and *in vitro* studies, the bioreactor offers several advantages as a tool for risk assessment of EDCs. *In vitro* gut-like culture systems, as used in the present study, are suitable for testing several conditions in parallel. It provides a method for pre-screening and thus supports the replacement of animal testing (46). By using a standardized model of the microbiota, a high degree of reproducibility could be achieved (26). Although not identical, the composition of the SIHUMIx consortium was quite similar after eight days of cultivation in the different bioreactor containers, as shown in the present and previous studies (26). In contrast, complex natural microbial consortia can behave remarkably differently *in vitro*, even under the same culture conditions and despite being derived from the same source (47). In humans, *in vitro* gut-like models are currently the only method available to directly study the effects of EDCs on the human gut microbiota. In particular, the urgent need for such studies is increasingly emphasized by the scientific and political community (48–50).

The commensal microbiota is key to maintaining intestinal homeostasis. It plays an important role in teaching the immune system to avoid inflammatory responses to harmless antigens, while providing defense against pathogens (51). Dysbiosis, in turn, can lead to inappropriate imprinting of the immune system, resulting in adverse health outcomes (5). Since EDCs have the potential to disrupt the microbiota, changing composition and metabolites, we hypothesized that exposure to BPX and/or PFAS may affect the immunomodulatory properties of the microbial community. To address this question, chemically treated SIHUMIx were co-cultured together with human PBMCs. As PBMCs contain different immune cell types, the assay mimics both the direct stimulation of immune cells by contact with microbial content and the interactions between different immune cell types, i.e. between antigen-presenting cells and effector immune cells. To overcome differences between bioreactors, we followed the activation course of immune cells after stimulation with microbiota samples from the same bioreactor, comparing the immune cells stimulated with EDC-treated SIHUMIx with those stimulated with untreated SIHUMIx (day 8 run) from the same bioreactor for each condition (ethanol, BPX, PFAS, BPX+PFAS). As expected, the most pronounced effect was observed on MAIT cells after incubation with SIHUMIx treated with the mixtures of BPX and PFAS, resulting in an overall reduced activation.

Several mechanisms may contribute to the alteration of the immune activation potential of SIHUMIX after EDC exposure. First, the diversity of the gut microbiota itself appears to determine MAIT cell activation. In a previous study, we found that a high microbial diversity was associated with reduced MAIT cell activation *in vitro* (52). A possible explanation could be that the uptake of riboflavin by certain bacteria exceeds the total production of this metabolite in complex communities (52). However, as we could not detect a significant effect of EDC exposure on riboflavin production by SIHUMIx, other mechanisms may be more likely to be responsible for the altered immunostimulatory potential observed in the present study. In addition to riboflavin, other metabolites have been shown to influence MAIT cell activation. For example, folate metabolites produced by the microbiota have an inhibitory effect on MAIT cells (22, 23). Furthermore, lactate has been shown to dampen MAIT cell activation after stimulation with *Staphylococcus aureus* cell-free supernatant (53). Therefore, detailed analysis of the metabolites produced by EDC-treated SIHUMIx may help to elucidate possible mechanisms by which EDCs affect the immunostimulatory potential of bacteria.

To get a comprehensive overview of the behavior of different immune populations after stimulation with chemically treated SIHUMIx, we performed t-SNE analysis which allows to identify rare or even unknown populations and to compare the expression patterns of cell and activation markers between different experimental groups (54). The expression of activation markers on several populations, including MAIT cells, NKT cells, and γδ T cells after stimulation with microbes, appeared to be dependent on the chemical treatment of SIHUMIx. This suggest that environmental chemicals may not only affect immune cells directly, but also indirectly via the gut microbiota. In particular, the activation status of MAIT cells was sensitive to the chemical treatment of SIHUMIx. Using t-SNE analysis, we were able to distinguish between different MAIT cell populations, namely CD8^+^ MAIT cells and DN MAIT cells. The latter was not measurable by manual gating due to very low cell numbers of this population, illustrating the power of t-SNE analysis to detect and visualize rare cell populations. As EDC-treatment – particularly the mixture of BPX and PFAS - appeared to remarkably alter the microbial properties to stimulate this rare cell population, it may be worth focusing on DN MAIT cells in future studies. These cells are functionally distinct from CD8^+^ MAIT cells, although the biological role of both populations is not yet fully understood (55). Furthermore, DN MAIT cells are the predominant MAIT cell population in mucosal tissues, but only a small subset in human peripheral blood (56). Therefore, immune cells at the epithelial barrier – where host-microbe interactions primarily occur – would be the more accurate model to focus on this immune cell type, but they are difficult to access.

In summary, we have developed a test system to study the effects of EDCs (and other chemicals) on microbiome-immune interactions *in vitro* on a large scale and without recourse to animal experiments. Another advantage of this model is that the effects can be analyzed at the level of specific immune cell subtypes, offering the possibility of identifying cells that are most sensitive to chemical effects. Furthermore, the model can be extended, in particular by using multi-omics approaches, such as metabolomics of the bacterial consortium and transcriptomics of the respective immune cells, providing mechanistic insights into the chemical-microbiome-immune interaction triangle. Future studies using our model may therefore increase the knowledge of how EDCs and their mixtures indirectly affect the immune system via altered microbiota.

## Data availability statement

The datasets presented in this study can be found in online repositories. The names of the repository/repositories and accession number(s) can be found below: PXD048273 (ProteomeXchange).

## Ethics statement

The studies involving humans were approved by Ethics Committee of the University of Leipzig (Ref. 079-15-09032015). The studies were conducted in accordance with the local legislation and institutional requirements. The participants provided their written informed consent to participate in this study.

## Author contributions

FF: Conceptualization, Data curation, Formal analysis, Investigation, Visualization, Writing – original draft, Writing – review & editing. AP: Methodology, Supervision, Validation, Visualization, Writing – review & editing. SR: Investigation, Methodology, Writing – review & editing. AA: Investigation, Writing – review & editing. VC-M: Investigation, Writing – review & editing. S-BH: Investigation, Visualization, Writing – review & editing. MvB: Funding acquisition, Resources, Writing – review & editing. UR-K: Investigation, Methodology, Writing – review & editing. NJ: Methodology, Supervision, Writing – review & editing. AZ: Funding acquisition, Resources, Writing – review & editing. GH: Conceptualization, Supervision, Writing – review & editing.
