# Supplementary material for: An in vitro model system for testing chemical effects on microbiome-immune interactions – examples with BPX and PFAS mixtures
Source: Front Immunol. 2024 Jun 17;15:1298971. doi: 10.3389/fimmu.2024.1298971 (PMC11215145; doi:10.3389/fimmu.2024.1298971)
Supplement: Supplementary file 1 [file DataSheet_1.docx]

***Supplementary Material***


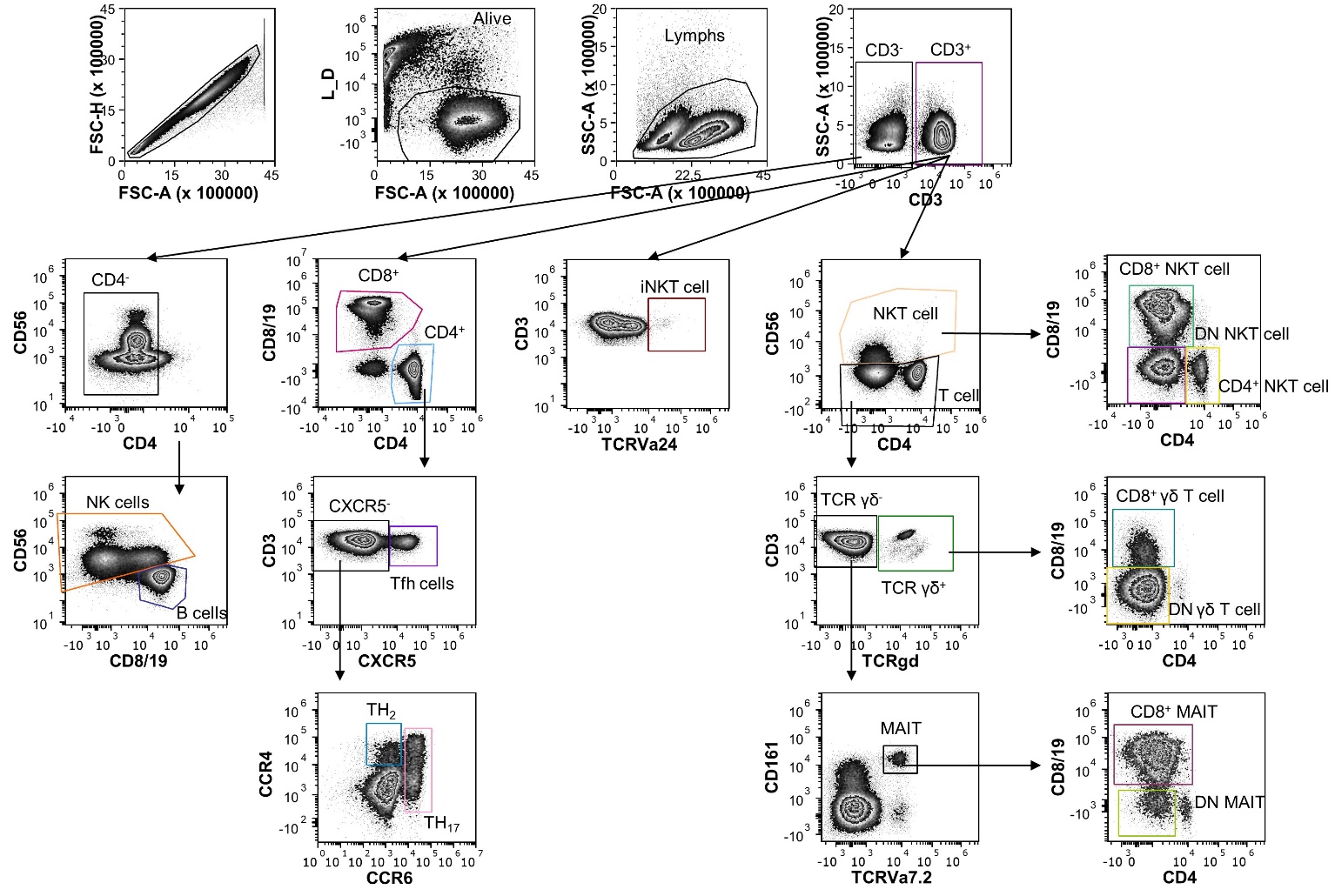


**Figure S 1. Gating Strategy**.


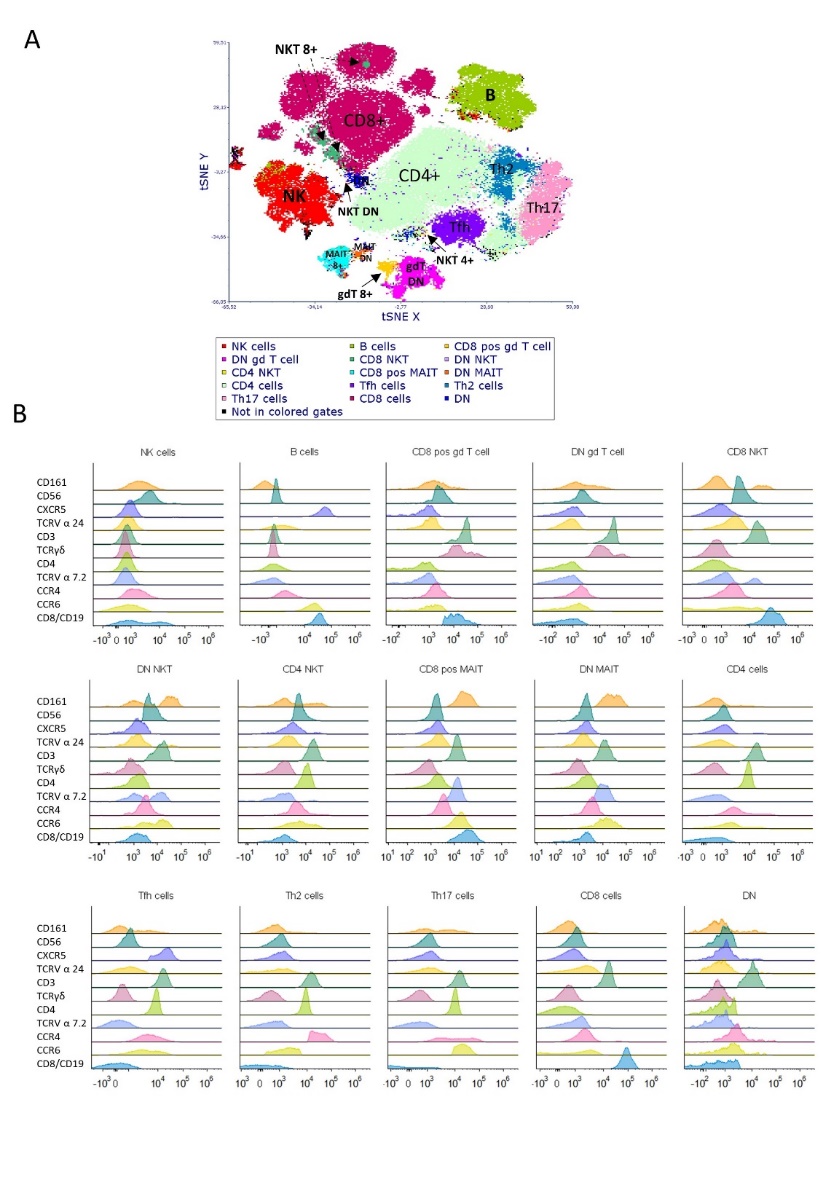


**Figure S 2. Population gating and characterization of human immune cells stimulated with EDC-treated SIHUMIx.** Human PBMC were stimulated for 6 hours in vitro with fixed SIHUMIx pre-exposed (bioreactor run day 10) to BPX (22 µM) and/or PFAS (4 µM) or ethanol as solvent control for 24 hours. The expression of indicated cell surface markers was measured by flow cytometry and analyzed via t-SNE. (A) The identification of distinct population was guided by the expression of cell surface markers. (B) The expression of cell surface markers (fluorescence intensity) is shown for individual populations. Abbreviations: DN, double negative for CD4/CD8 marker; MAIT 8+, for CD8 positive MAIT cells; NKT 8+ for CD8 positive NKT cells; gdt 8+ for CD8 positive gd T cells


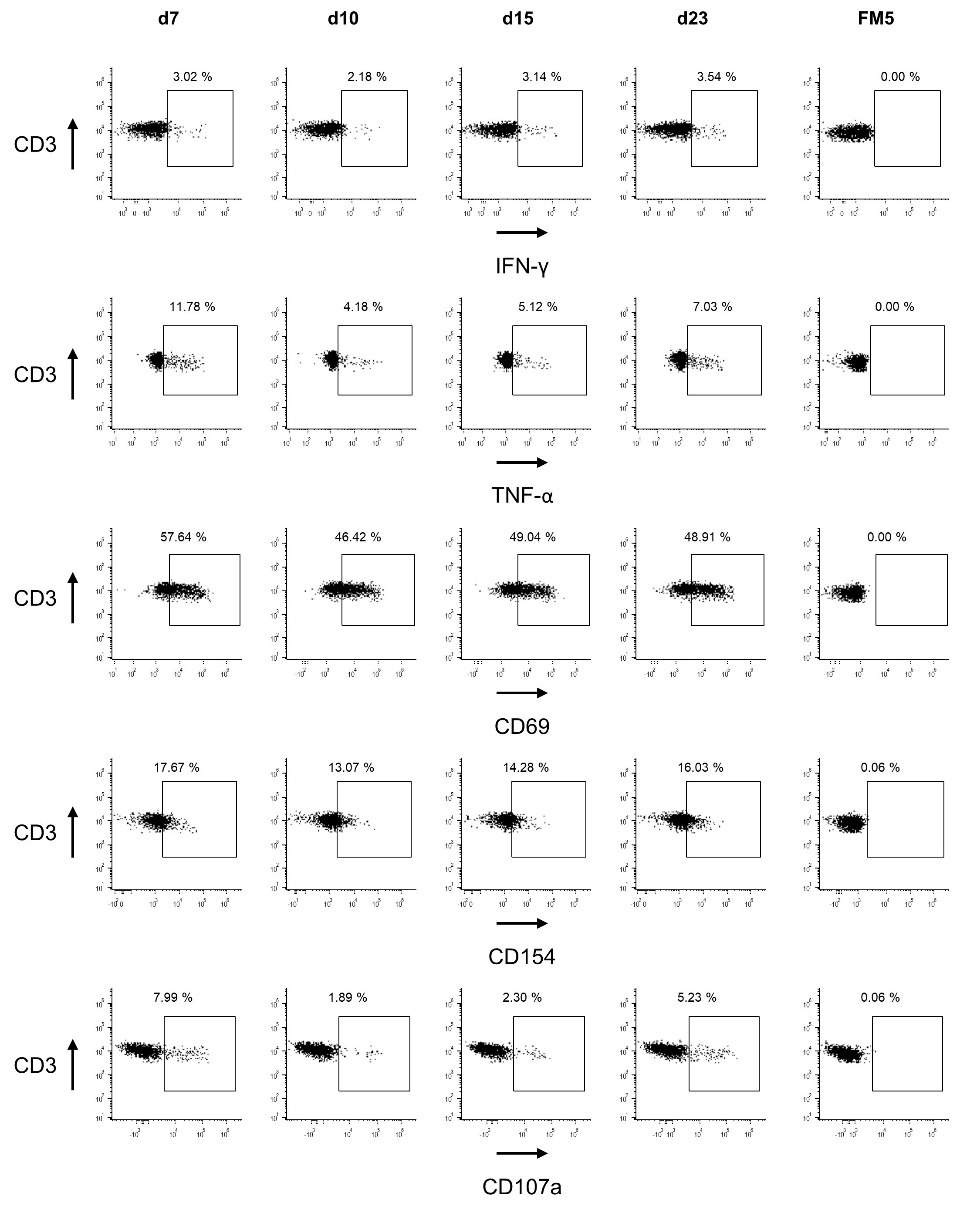


**Figure S 3. Activation of human CD8+ MAIT cells stimulated with BPX+PFAS-treated SIHUMIx.** Representative flow cytometry plots showing the expression of indicated activation markers in human CD8^+^ MAIT cells after 6 hours in vitro stimulation of PBMC with BPX+PFAS-treated SIHUMIx sampled at bioreactor run day 7, 10, 15 and 23. Right column shows representative flow cytometry plots for fluorescence minus five (FM5) controls.

**Table S 1. Antibodies used in the present study.**

| **Antibody** | **Fluorochrome** | **Company** | **Catalogue no.** | **Clone** | **Dilution** | **Concentration** |
| --- | --- | --- | --- | --- | --- | --- |
| γδTCR | PE | BioLegend | 331210 | B1 | 100 | 4 µg/ml |
| CXCR5 | BV711 | BioLegend | 356934 | J252D4 | 100 | 1 µg/ml |
| CD161 | BV421 | BioLegend | 339914 | HP-3G10 | 100 | 1 µg/ml |
| CD56 | BV510 | BioLegend | 318340 | HCD56 | 200 | 0.5 µg/ml |
| TCRVa24 | BV785 | BioLegend | 342932 | 6B11 | 100 | 0.5 µg/ml |
| CD3 | SparkBlue550 | BioLegend | 344.852 | SK7 | 100 | 2 µg/ml |
| CD4 | PerCP | BioLegend | 344.624 | SK3 | 100 | 0.25 µg/ml |
| TCRVa7.2 | PerCP-Cy5.5 | BioLegend | 351710 | 3C10 | 100 | 2 µg/ml |
| CCR4 | PE-Cy7 | BioLegend | 359410 | I.291H4 | 200 | 1 µg/ml |
| CCR10 | APC | Miltenyi | 130-104-821 | REA326 | 50 | NA |
| CCR6 | APC-Cy7 | BioLegend | 353432 | G034E3 | 200 | 0.5 µg/ml |
| CD8 | APC-Fire810 | BioLegend | 344764 | SK1 | 400 | 0.125 mg/ml |
| CD19 | APC-Fire810 | BioLegend | 302272 | HIB19 | 200 | 0.25 µg/ml |
| IFNg | PacificBlue | BioLegend | 502522 | 4S.B3 | 200 | 2.5 µg/ml |
| TNF | BV605 | BioLegend | 502936 | MAb11 | 100 | 1 µg/ml |
| CD69 | FITC | BioLegend | 310904 | FN50 | 100 | 1 µg/ml |
| CD154 | PE-Dazzle594 | BioLegend | 310840 | 24-31 | 100 | 1.5 µg/ml |

NA, no information available from the manufacturer

**Table S2: Bisphenol test concentrations**

Table S2 Characteristics of bisphenols used in the study

| Chemical | M [g/mol] | Solvent |
| --- | --- | --- |
| Bisphenol F | 200.24 | Ethanol |
| Bisphenol S | 250.27 | Ethanol |

Exposure concentrations of bisphenol F and bisphenol S were calculated according to a 70 kg person with a daily intake of 4 µg/kg or 5 µg/kg. The average stool weight per day (123.6 g) (Rendtorff and Kashgarian, 1967) was included in the calculations.

4 µg/kg body weight:

$$\frac{(4 \mu g/kg*70 kg)}{123.6 g}=22.65{\mu g}/g$$

5 µg/kg body weight:

$$\frac{(5 \mu g/kg*70 kg)}{123.6 g}=28.32 {\mu g}/g$$

We converted µg/g of stool into mg/ml, due to our liquid cultivation in bioreactors. This led to 0.0226 mg/ml and 0.0283 mg/ml for 4 µg/kg and 5 µg/kg, respectively. Molar concentrations (µM/L) were calculated for each bisphenol according to their specific molar mass. To achieve the same molar concentrations in our mixture, we calculated bisphenol F with 0.0226 mg/ml, corresponding to 11 µM. For bisphenol S we achieved 0.0283 mg/ml, which corresponds to 11 µM.

References

Rendtorff RC, Kashgarian M. Stool patterns of healthy adult males. *Dis Colon*

*Rectum*. (1967) 10:222–8. doi: 10.1007/BF02617184
